# 2-Hy­droxy-*N*-(4-meth­oxy­benz­yl)-4-nitro­anilinium chloride

**DOI:** 10.1107/S1600536811029552

**Published:** 2011-07-30

**Authors:** Raouf Boulcina, Boubakeur Fantazi, Sofiane Bouacida, Thierry Roisnel, Abdelmadjid Debache

**Affiliations:** a Laboratoire des Produits Naturels d’origine Végétale et de Synthèse Organique, PHYSYNOR, Université Mentouri-Constantine, 25000 Constantine, Algeria; b Unité de Recherche de Chimie de l’Environnement et Moléculaire Structurale, CHEMS, Université Mentouri-Constantine, 25000 Algeria; c Centre de Difractométrie X, UMR 6226 CNRS Unité Sciences Chimiques de Rennes, Université de Rennes I, 263 Avenue du Général Leclerc, 35042 Rennes, France

## Abstract

The crystal structure of the title compound, C_14_H_15_N_2_O_4_^+^·Cl^−^, can be described as being composed of layers containing both cations and anions that are staggered along [010]. Two types of the hydrogen bonds are observed, *viz.* cation–anion and cation–cation. The chloride anions are acceptors of the strong hydrogen bonds donated by the secondary amine and the hy­droxy groups. The packing is also stabilized by weak C—H⋯O inter­molecular hydrogen bonds. An intra­molecular N—H⋯O inter­action also occurs.

## Related literature

For the preparation of amines, see: Apodaca & Xiao (2001 ▶); Baxter & Reitz (2002 ▶); Salvatore *et al.* (2002 ▶); Sato *et al.* (2004 ▶). For applications of amines, see: Bergeron *et al.* (1997 ▶); Seayad *et al.* (2002 ▶). For background to hydrogen bonding, see: Desiraju (2003 ▶); Dorn *et al.* (2005 ▶) and for hydrogen-bond motifs, see: Etter *et al.* (1990 ▶).
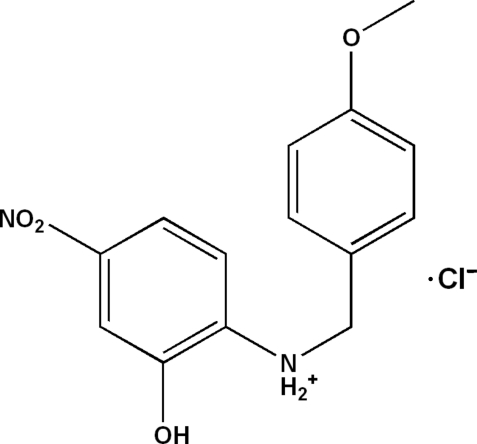


## Experimental

### 

#### Crystal data

C_14_H_15_N_2_O_4_^+^·Cl^−^
*M* *_r_* = 310.73
Monoclinic, 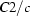

*a* = 32.1166 (9) Å
*b* = 7.4888 (2) Å
*c* = 13.0907 (4) Å
β = 108.655 (2)°
*V* = 2983.09 (15) Å^3^
*Z* = 8
Mo *K*α radiation
μ = 0.27 mm^−1^
*T* = 100 K
0.18 × 0.12 × 0.06 mm

#### Data collection

Bruker APEXII CCD area-detector diffractometer
Absorption correction: multi-scan (*SADABS*; Sheldrick, 2002 ▶) *T*_min_ = 0.784, *T*_max_ = 0.984
11129 measured reflections
3359 independent reflections
2290 reflections with *I* > 2σ(*I*)
*R*_int_ = 0.049

#### Refinement

*R*[*F* ^2^ > 2σ(*F* ^2^)] = 0.045
*wR*(*F* ^2^) = 0.079
*S* = 1.35
3359 reflections
191 parameters
H-atom parameters constrained
Δρ_max_ = 0.32 e Å^−3^
Δρ_min_ = −0.25 e Å^−3^

### 

Data collection: *APEX2* (Bruker, 2003 ▶); cell refinement: *SAINT* (Bruker, 2003 ▶); data reduction: *SAINT*; program(s) used to solve structure: *SIR2002* (Burla *et al.*, 2003 ▶); program(s) used to refine structure: *SHELXL97* (Sheldrick, 2008 ▶); molecular graphics: *ORTEP-3 for Windows* (Farrugia, 1997 ▶) and *DIAMOND* (Brandenburg & Berndt, 2001 ▶); software used to prepare material for publication: *WinGX* (Farrugia, 1999 ▶).

## Supplementary Material

Crystal structure: contains datablock(s) global, I. DOI: 10.1107/S1600536811029552/fb2237sup1.cif

Structure factors: contains datablock(s) I. DOI: 10.1107/S1600536811029552/fb2237Isup2.hkl

Supplementary material file. DOI: 10.1107/S1600536811029552/fb2237Isup3.cml

Additional supplementary materials:  crystallographic information; 3D view; checkCIF report

## Figures and Tables

**Table 1:** Hydrogen-bond geometry (Å, °)

*D*—H⋯*A*	*D*—H	H⋯*A*	*D*⋯*A*	*D*—H⋯*A*
O1—H1⋯Cl1	0.84	2.16	2.9950 (13)	174
N10—H10*A*⋯O1	0.92	2.22	2.652 (2)	108
N10—H10*A*⋯Cl1^i^	0.92	2.30	3.1082 (17)	146
N10—H10*B*⋯Cl1^ii^	0.92	2.23	3.0518 (15)	149
C8—H8⋯O5^ii^	0.93	2.58	3.273 (2)	132
C14—H14⋯O6^iii^	0.93	2.48	3.388 (3)	165

Symmetry codes: (i) 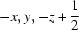; (ii) 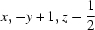; (iii) 
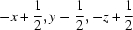
.

**Fig. 1.**
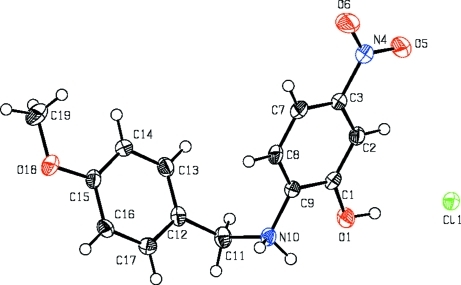
The title molecule (Farrugia, 1997) with the atomic labelling scheme. The displacement parameters are drawn at the 50% probability level.

**Fig. 2.**
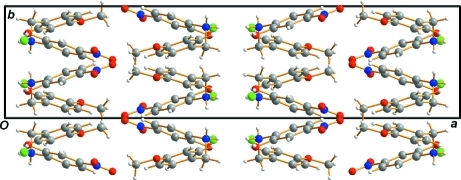
The packing of the title structure viewed down the c axis (Brandenburg & Berndt, 2001). Cl is shown in green, N in blue and C in grey.

**Fig. 3.**
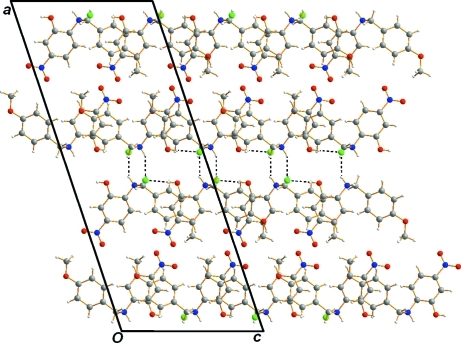
A section of the title structure showing the hydrogen bonds N—H···Cl and O—H···Cl as dashed lines (Brandenburg & Berndt, 2001). Cl is shown in green, N in blue and C in grey.

**Fig. 4.**
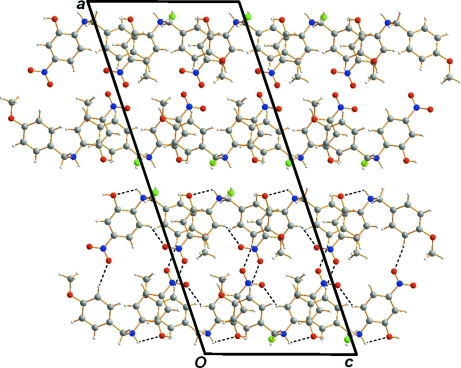
A section of the title structure showing intermolecular hydrogen bond N—H···O and weak C—H···O interactions as dashed lines (Brandenburg & Berndt, 2001). Cl is shown in green, N in blue and C in grey.

**Fig. 5.**
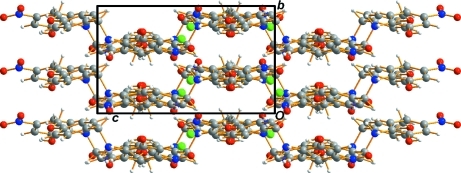
The packing of the title structure viewed down the a axis (Brandenburg & Berndt, 2001). Cl is shown in green, N in blue and C in grey.

## supplementary crystallographic information

### Comment

Amines are one of the most important classes of biologically active compounds of natural origin. They are also widely used in the chemical industry as basic intermediates for preparation of *e.g.* fine chemicals, pharmaceuticals and agrochemicals (Seayad *et al.*, 2002).

Due to their biological properties, amines have played important role in chemotherapeutic treatment of different diseases (Bergeron *et al.*, 1997).

Alkylation of the secondary amines with alkyl halides is the most straightforward method for the synthesis of the tertiary amines (Salvatore *et al.*, 2002). Reductive amination of aldehydes andketones is a powerful tool for the synthesis of amines. This approach is extensively used for rapid access to diverse sets of amines (Sato *et al.*, 2004; Baxter *et al.*, 2002; Apodaca *et al.*, 2001).

The synthetic route we envisioned for preparation of the title compounds consists of a one-step reductive amination of aromatic aldehydes with primary amine at acid conditions (pH = 4-5). We found that this could be efficiently conducted in methanol at room temperature using the excess of reductive agent (NaBH_3_CN). Under these conditions 2-(4-methoxybenzylamino)-5-nitrophenol was cleanly obtained in very good chemical yield (85%).

Fig. 1 shows the title molecule. The two benzene rings contain the interplanar angle equal to  35.35 (6)°. In the crystal packing, the important role play the hydrogen bonds. In the title structure, two types of hydrogen bonds are present, interconnecting the cations with the anions as well as mutually the cations. The chloride anions are involved as acceptors in the strong hydrogen bonds (Desiraju, 2003; Dorn *et al.* 2005) with the secondary amine and the hydroxy group stemming from the cation (Tab. 1), *i.e.* in [O—H···Cl^-^ and N—H···Cl^-^] hydrogen bonds interactions.

The layers staggered along the *b* axis can be discerned in the crystal structure (Fig. 2). Each layer contains dimers composed of the cations and Cl^-^. These dimers are situated about the crystallographic two-fold axes. The dimers form the motifs R^2^_4_(14) (Etter *et al.*, 1990)  with a pair of chains O1-H1···Cl1···H10*a*-N10-C1-C9 (Fig. 3). Moreover, the dimers are interconnected with those in the adjacent layer by another pair of the hydrogen bonds Cl1···H10*b*-N10-H10*a* with the graph set motif R^2^_4_(8) (Fig. 3). The latter motifs are situated about the crystallographic inversion centres. The packing is also stabilized by weak N—H···O (intramolecular) and C—H···O (intermolecular) interactions (Fig. 4, Tab. 1). Fig. 5 shows the projection of the structure along the *a* axis.

### Experimental

To the solution of 4-methoxybenzaldehyde (2 mmol) in dry methanol (10 ml) 5-nitro-2-aminophenol (2 mmol) was added and acidified by concentrated HCl until pH = 6. After vigorous stirring for 2 h at room temperature, NaBH_3_CN (6 mmol) was added. On completion of the reaction, as indicated by thin layer chromatography (ethyl acetate/hexane: 1/3 as eluent), the excess of the hydride was carefully destroyed by slow addition of 20 ml of cold water. The mixture was left for several hours and the resulting precipitate was filtered off, washed with water, then with ethanol and with hexane. 2-(4-methoxybenzylamino)-5-nitrophenol was identified by IR, ^1^H and ^13^C NMR spectroscopies. Colourless prismatic crystals (0.06×0.12×0.18 mm) of the title structure were obtained by slow crystallization from the aqueous solution with pH = 5.5.

### Refinement

Approximate positions for all the H atoms were first obtained from the difference electron density map. However, the H atoms were situated into idealized positions and the H-atoms have been refined within the riding atom approximation. The applied constraints were as follow: C_aryl_—H_aryl_ = 0.95 Å; C_methyl_—H_methyl_ = 0.98 Å; C_methylene_—H_methylene_ = 0.99Å and N_sec.amine_—H_sec.amine_ = 0.84 Å. The idealized methyl group was allowed to rotate about the C—C bond during the refinement by application of the command AFIX 137 in *SHELXL97* (Sheldrick, 2008). *U*_iso_(H_methyl_/_hydroxy_) = 1.5*U*_eq_(C_methyl_/O_hydroxy_) or *U*_iso_(H_aryl_/_methylene_/_sec. amine_) = 1.2*U*_eq_(C_aryl_/C_methylene_/N_sec. amine_).

### Crystal data

**Table:**

C_14_H_15_N_2_O_4_^+^·Cl^−^	*F*(000) = 1296
*M**_r_* = 310.73	*D*_x_ = 1.384 Mg m^−^^3^
Monoclinic, *C*2/*c*	Mo *K*α radiation, λ = 0.71073 Å
Hall symbol: -C 2yc	Cell parameters from 1996 reflections
*a* = 32.1166 (9) Å	θ = 2.7–25.9°
*b* = 7.4888 (2) Å	µ = 0.27 mm^−^^1^
*c* = 13.0907 (4) Å	*T* = 100 K
β = 108.655 (2)°	Prism, colourless
*V* = 2983.09 (15) Å^3^	0.18 × 0.12 × 0.06 mm
*Z* = 8	

### Data collection

**Table:**

Bruker APEXII CCD area-detector diffractometer	2290 reflections with *I* > 2σ(*I*)
graphite	*R*_int_ = 0.049
φ and ω scans	θ_max_ = 27.5°, θ_min_ = 3.1°
Absorption correction: multi-scan (*SADABS*; Sheldrick, 2002)	*h* = −41→36
*T*_min_ = 0.784, *T*_max_ = 0.984	*k* = −9→8
11129 measured reflections	*l* = −16→13
3359 independent reflections	

### Refinement

**Table:**

Refinement on *F*^2^	Primary atom site location: structure-invariant direct methods
Least-squares matrix: full	Secondary atom site location: difference Fourier map
*R*[*F*^2^ > 2σ(*F*^2^)] = 0.045	Hydrogen site location: difference Fourier map
*wR*(*F*^2^) = 0.079	H-atom parameters constrained
*S* = 1.35	*w* = 1/[σ^2^(*F*_o_^2^) + (0.010*P*)^2^] where *P* = (*F*_o_^2^ + 2*F*_c_^2^)/3
3359 reflections	(Δ/σ)_max_ = 0.001
191 parameters	Δρ_max_ = 0.32 e Å^−^^3^
0 restraints	Δρ_min_ = −0.25 e Å^−^^3^
59 constraints	

### Special details

**Table:**

Geometry. All e.s.d.'s (except the e.s.d. in the dihedral angle between two l.s. planes) are estimated using the full covariance matrix. The cell e.s.d.'s are taken into account individually in the estimation of e.s.d.'s in distances, angles and torsion angles; correlations between e.s.d.'s in cell parameters are only used when they are defined by crystal symmetry. An approximate (isotropic) treatment of cell e.s.d.'s is used for estimating e.s.d.'s involving l.s. planes.
Refinement. Refinement of *F*^2^ against ALL reflections. The weighted *R*-factor *wR* and goodness of fit *S* are based on *F*^2^, conventional *R*-factors *R* are based on *F*, with *F* set to zero for negative *F*^2^. The threshold expression of *F*^2^ > σ(*F*^2^) is used only for calculating *R*-factors(gt) *etc*. and is not relevant to the choice of reflections for refinement. *R*-factors based on *F*^2^ are statistically about twice as large as those based on *F*, and *R*- factors based on ALL data will be even larger.

### Fractional atomic coordinates and isotropic or equivalent isotropic displacement parameters (Å^2^)

**Table:**

	*x*	*y*	*z*	*U*_iso_*/*U*_eq_	
O1	0.05089 (4)	0.24108 (16)	0.25570 (10)	0.0282 (4)	
H1	0.0481	0.2513	0.317	0.042*	
O5	0.19661 (4)	0.3946 (2)	0.52794 (12)	0.0450 (4)	
O6	0.23649 (5)	0.4782 (2)	0.43081 (12)	0.0596 (5)	
O18	0.16637 (4)	0.13654 (17)	−0.26241 (11)	0.0333 (4)	
N4	0.20173 (6)	0.4259 (2)	0.44038 (15)	0.0365 (5)	
N10	0.05796 (5)	0.31371 (19)	0.06366 (12)	0.0219 (4)	
H10A	0.0329	0.3166	0.0834	0.026*	
H10B	0.0562	0.4062	0.0162	0.026*	
C1	0.09069 (6)	0.3047 (2)	0.25869 (16)	0.0223 (5)	
C2	0.12573 (6)	0.3321 (2)	0.35182 (16)	0.0250 (5)	
H2	0.1233	0.3074	0.4194	0.03*	
C3	0.16445 (6)	0.3973 (3)	0.34105 (16)	0.0259 (5)	
C7	0.16978 (6)	0.4387 (3)	0.24313 (16)	0.0289 (5)	
H7	0.1963	0.4833	0.2395	0.035*	
C8	0.13458 (6)	0.4118 (2)	0.15093 (16)	0.0247 (5)	
H8	0.1369	0.439	0.0837	0.03*	
C9	0.09576 (6)	0.3441 (2)	0.15932 (15)	0.0207 (5)	
C11	0.05879 (6)	0.1389 (2)	0.00555 (16)	0.0273 (5)	
H11A	0.068	0.044	0.0584	0.033*	
H11B	0.0293	0.1116	−0.0407	0.033*	
C12	0.08902 (6)	0.1425 (2)	−0.06154 (16)	0.0232 (5)	
C13	0.13234 (6)	0.0854 (2)	−0.02110 (16)	0.0267 (5)	
H13	0.1432	0.0459	0.0499	0.032*	
C14	0.15987 (6)	0.0858 (2)	−0.08398 (16)	0.0264 (5)	
H14	0.1891	0.0507	−0.0548	0.032*	
C15	0.14329 (6)	0.1393 (2)	−0.19111 (16)	0.0236 (5)	
C16	0.10009 (6)	0.1979 (2)	−0.23310 (15)	0.0245 (5)	
H16	0.089	0.2353	−0.3045	0.029*	
C17	0.07379 (6)	0.2001 (2)	−0.16807 (16)	0.0248 (5)	
H17	0.045	0.2412	−0.1962	0.03*	
C19	0.21172 (6)	0.0849 (3)	−0.22251 (18)	0.0447 (6)	
H19A	0.2139	−0.0351	−0.1955	0.067*	
H19B	0.2273	0.1641	−0.1654	0.067*	
H19C	0.2243	0.0912	−0.2799	0.067*	
Cl1	0.042616 (15)	0.30709 (6)	0.47404 (4)	0.02477 (14)	

### Atomic displacement parameters (Å^2^)

**Table:**

	*U*^11^	*U*^22^	*U*^33^	*U*^12^	*U*^13^	*U*^23^
O1	0.0289 (8)	0.0383 (9)	0.0212 (8)	−0.0058 (6)	0.0133 (7)	0.0010 (6)
O5	0.0345 (9)	0.0751 (12)	0.0242 (9)	0.0038 (8)	0.0079 (7)	−0.0032 (8)
O6	0.0226 (9)	0.1194 (16)	0.0372 (10)	−0.0117 (9)	0.0102 (8)	−0.0126 (10)
O18	0.0293 (8)	0.0455 (10)	0.0306 (9)	0.0039 (7)	0.0174 (7)	−0.0011 (7)
N4	0.0260 (11)	0.0543 (13)	0.0292 (12)	0.0056 (9)	0.0087 (9)	−0.0083 (10)
N10	0.0229 (9)	0.0259 (10)	0.0201 (9)	−0.0018 (7)	0.0112 (7)	−0.0006 (8)
C1	0.0221 (11)	0.0214 (11)	0.0265 (12)	0.0034 (9)	0.0120 (9)	0.0004 (9)
C2	0.0275 (12)	0.0290 (13)	0.0200 (11)	0.0072 (9)	0.0096 (9)	0.0017 (9)
C3	0.0215 (11)	0.0328 (13)	0.0230 (12)	0.0054 (9)	0.0066 (9)	−0.0049 (10)
C7	0.0209 (11)	0.0378 (14)	0.0309 (13)	0.0003 (9)	0.0125 (10)	−0.0048 (10)
C8	0.0268 (11)	0.0309 (13)	0.0211 (11)	0.0013 (9)	0.0140 (9)	−0.0004 (9)
C9	0.0205 (10)	0.0206 (12)	0.0212 (11)	0.0015 (8)	0.0072 (9)	−0.0018 (9)
C11	0.0337 (12)	0.0237 (12)	0.0263 (12)	−0.0035 (9)	0.0121 (10)	−0.0044 (9)
C12	0.0285 (12)	0.0216 (12)	0.0217 (11)	−0.0027 (9)	0.0112 (9)	−0.0035 (9)
C13	0.0321 (12)	0.0284 (13)	0.0192 (11)	0.0032 (10)	0.0073 (9)	−0.0007 (9)
C14	0.0220 (11)	0.0317 (13)	0.0246 (12)	0.0039 (9)	0.0060 (9)	−0.0026 (10)
C15	0.0254 (11)	0.0257 (12)	0.0228 (12)	−0.0031 (9)	0.0120 (9)	−0.0046 (9)
C16	0.0276 (11)	0.0276 (12)	0.0171 (11)	0.0006 (9)	0.0053 (9)	0.0017 (9)
C17	0.0214 (11)	0.0267 (12)	0.0253 (12)	−0.0014 (9)	0.0064 (9)	−0.0019 (10)
C19	0.0297 (13)	0.0644 (17)	0.0467 (16)	0.0054 (12)	0.0218 (12)	−0.0007 (13)
Cl1	0.0245 (3)	0.0286 (3)	0.0224 (3)	0.0021 (2)	0.0091 (2)	−0.0001 (2)

### Geometric parameters (Å, °)

**Table:**

O1—C1	1.353 (2)	C8—C9	1.382 (2)
O1—H1	0.84	C8—H8	0.93
O5—N4	1.231 (2)	C11—C12	1.503 (2)
O6—N4	1.2268 (19)	C11—H11A	0.97
O18—C15	1.365 (2)	C11—H11B	0.97
O18—C19	1.434 (2)	C12—C13	1.389 (2)
N4—C3	1.475 (2)	C12—C17	1.390 (3)
N10—C9	1.456 (2)	C13—C14	1.387 (2)
N10—C11	1.519 (2)	C13—H13	0.93
N10—H10A	0.9201	C14—C15	1.390 (3)
N10—H10B	0.9201	C14—H14	0.93
C1—C2	1.385 (2)	C15—C16	1.390 (2)
C1—C9	1.393 (2)	C16—C17	1.378 (2)
C2—C3	1.385 (2)	C16—H16	0.93
C2—H2	0.93	C17—H17	0.93
C3—C7	1.381 (3)	C19—H19A	0.96
C7—C8	1.379 (2)	C19—H19B	0.96
C7—H7	0.93	C19—H19C	0.96
			
C1—O1—H1	109.3	C12—C11—H11A	108.9
C15—O18—C19	117.73 (15)	N10—C11—H11A	108.9
O6—N4—O5	123.56 (18)	C12—C11—H11B	108.9
O6—N4—C3	117.71 (18)	N10—C11—H11B	108.9
O5—N4—C3	118.73 (17)	H11A—C11—H11B	107.7
C9—N10—C11	114.97 (13)	C13—C12—C17	117.77 (18)
C9—N10—H10A	108.5	C13—C12—C11	121.83 (18)
C11—N10—H10A	108.5	C17—C12—C11	120.38 (17)
C9—N10—H10B	108.5	C14—C13—C12	121.61 (18)
C11—N10—H10B	108.5	C14—C13—H13	119.2
H10A—N10—H10B	107.5	C12—C13—H13	119.2
O1—C1—C2	124.88 (17)	C13—C14—C15	119.21 (18)
O1—C1—C9	116.05 (17)	C13—C14—H14	120.4
C2—C1—C9	119.07 (17)	C15—C14—H14	120.4
C3—C2—C1	117.78 (18)	O18—C15—C16	115.21 (17)
C3—C2—H2	121.1	O18—C15—C14	124.67 (17)
C1—C2—H2	121.1	C16—C15—C14	120.11 (18)
C7—C3—C2	123.74 (19)	C17—C16—C15	119.42 (18)
C7—C3—N4	118.62 (17)	C17—C16—H16	120.3
C2—C3—N4	117.63 (18)	C15—C16—H16	120.3
C3—C7—C8	117.97 (18)	C16—C17—C12	121.83 (18)
C3—C7—H7	121	C16—C17—H17	119.1
C8—C7—H7	121	C12—C17—H17	119.1
C9—C8—C7	119.44 (18)	O18—C19—H19A	109.5
C9—C8—H8	120.3	O18—C19—H19B	109.5
C7—C8—H8	120.3	H19A—C19—H19B	109.5
C8—C9—C1	121.97 (18)	O18—C19—H19C	109.5
C8—C9—N10	120.92 (17)	H19A—C19—H19C	109.5
C1—C9—N10	117.10 (16)	H19B—C19—H19C	109.5
C12—C11—N10	113.27 (14)		
			
O1—C1—C2—C3	179.67 (17)	C11—N10—C9—C8	82.5 (2)
C9—C1—C2—C3	−0.2 (3)	C11—N10—C9—C1	−98.47 (19)
C1—C2—C3—C7	0.9 (3)	C9—N10—C11—C12	−77.3 (2)
C1—C2—C3—N4	−179.87 (16)	N10—C11—C12—C13	93.1 (2)
O6—N4—C3—C7	−3.1 (3)	N10—C11—C12—C17	−88.4 (2)
O5—N4—C3—C7	177.68 (18)	C17—C12—C13—C14	0.3 (3)
O6—N4—C3—C2	177.70 (18)	C11—C12—C13—C14	178.75 (17)
O5—N4—C3—C2	−1.6 (3)	C12—C13—C14—C15	−2.1 (3)
C2—C3—C7—C8	−0.6 (3)	C19—O18—C15—C16	177.25 (17)
N4—C3—C7—C8	−179.78 (17)	C19—O18—C15—C14	−3.9 (3)
C3—C7—C8—C9	−0.5 (3)	C13—C14—C15—O18	−176.41 (17)
C7—C8—C9—C1	1.2 (3)	C13—C14—C15—C16	2.4 (3)
C7—C8—C9—N10	−179.87 (16)	O18—C15—C16—C17	178.03 (16)
O1—C1—C9—C8	179.28 (16)	C14—C15—C16—C17	−0.9 (3)
C2—C1—C9—C8	−0.8 (3)	C15—C16—C17—C12	−1.0 (3)
O1—C1—C9—N10	0.3 (2)	C13—C12—C17—C16	1.3 (3)
C2—C1—C9—N10	−179.82 (15)	C11—C12—C17—C16	−177.19 (17)

### Hydrogen-bond geometry (Å, °)

**Table:**

*D*—H···*A*	*D*—H	H···*A*	*D*···*A*	*D*—H···*A*
O1—H1···Cl1	0.84	2.16	2.9950 (13)	174
N10—H10A···O1	0.92	2.22	2.652 (2)	108
N10—H10A···Cl1^i^	0.92	2.30	3.1082 (17)	146
N10—H10B···Cl1^ii^	0.92	2.23	3.0518 (15)	149
C8—H8···O5^ii^	0.93	2.58	3.273 (2)	132
C14—H14···O6^iii^	0.93	2.48	3.388 (3)	165

Symmetry codes: (i) −*x*, *y*, −*z*+1/2; (ii) *x*, −*y*+1, *z*−1/2; (iii) −*x*+1/2, *y*−1/2, −*z*+1/2.
